# Evolutionary dynamics of endogenous densoviruses NS1 proteins reveal ancient codivergence with their Platyhelminth hosts

**DOI:** 10.1128/spectrum.03441-25

**Published:** 2026-02-27

**Authors:** Han Zhang, Juan Xu, Xiaodong Su, Qing Zhang, Hua Wang, Ping Wu, Xiao Ma, Wen Zhang, Chenglin Zhou

**Affiliations:** 1 Department of Laboratory Medicine, School of Medicine, Jiangsu University 12676, Zhenjiang, Jiangsu, China; 2 Clinical Laboratory Center, The Affiliated Taizhou People's Hospital of Nanjing Medical University 372209 , Taizhou, China; 3 Department of the Center for Disease Control and Prevention, Tianjun, China; 4 Department of Parasitic Disease Prevention and Control, Qinghai Institute of Endemic Disease Prevention and Control, Xining, China; Fudan University, Shanghai, China

**Keywords:** host-virus codivergence, evolutionary dynamics, Platyhelminth hosts

## Abstract

Endogenous viral elements serve as molecular fossils of ancient viral infections, providing valuable evidence for reconstructing the evolutionary history of modern viruses and their hosts. These genomic remnants also provide crucial benchmarks for calibrating viral evolutionary timelines. Here, our flanking sequence analysis reveals that densovirus NS1 sequences are chromosomally integrated in Platyhelminth genomes. Furthermore, their evolutionary dynamics reflect an ancient host-virus codivergence event dating to the Ediacaran-Cambrian transition (approximately 692 million years ago, MYA). Phylogenomic analyses delineate profound divergence between endogenous densovirus sequences in Platyhelminthes and exogenous Parvoviridae sequences, with distinct phylogenetic clustering supporting long-term endogenization. Structural alignments reveal significant global divergence (RMSD: 15.048–36.725 Å) yet striking local conservation in NS1 proteins, with the ATPase (RMSD: 0.271–1.756 Å) and effector domains (RMSD: 2.446–4.775 Å) remaining highly conserved across Platyhelminth hosts. The timescale phylogenetic tree inferred ancient divergence times of 233.51 MYA for *Schistosoma-Dicrocoelium* and 190.43 MYA for the *Clonorchis sinensis*, *Opisthorchis viverrini*, and *Paragonimus westermani* lineage. Distance matrix analysis revealed pronounced interspecific divergence and intraspecific conservation in NS1 sequences, consistent with a host-virus codivergence model. These findings reveal the preservation of replication-critical domains and the optimization of host-specific adaptation, providing molecular evidence for virus-parasite coevolution over geological timescales. Collectively, our findings reshape our understanding of virus-host coevolution and offer a framework to reconstruct paleoviral dynamics using endogenous “molecular fossils.”

**IMPORTANCE:**

This study provides evidence of densoviral endogenization in Platyhelminth hosts, advancing understanding of virus-host codivergence and offering a framework for reconstructing paleoviral dynamics using endogenous “molecular fossils.”

## INTRODUCTION

Densoviruses, belonging to the subfamily Densovirinae of the Parvoviridae, comprise small, non-enveloped viruses with linear, single-stranded DNA genomes ranging from 4 to 6 kilobases (kb) in size (1–3). The Parvoviridae is divided into three primary subfamilies: the Parvovirinae*,* which infect vertebrates; the Densovirinae, which primarily infect invertebrates; and the Hamaparvovirinae, which exhibits a broader host range, infecting both invertebrates and vertebrates (4, 5). The viruses are characterized by two gene cassettes. The first encodes nonstructural proteins (NS), which are critical for viral gene expression and DNA replication, while the second encodes structural capsid proteins (VP). Among the NS proteins, the multi-functional domain NS1 is the largest and most conserved, playing a pivotal role in viral replication. NS1 contains a superfamily 3 (SF3) helicase domain, which confers ATPase and helicase activities, as well as additional domains responsible for site-specific endonuclease activity and DNA binding (6, 7). These enzymatic functions are indispensable for viral genome replication and packaging.

Densoviruses are primarily known to infect invertebrate hosts, including insects, crustaceans, and echinoderms (8, 9). While Platyhelminthes are not considered typical hosts, emerging evidence suggests that certain densoviruses can infect these organisms. For instance, densovirus-like sequences have been identified in the transcriptome of *Echinococcus multilocularis*, with evidence of their genomic integration into *cestode* genomes (10). Similarly, fragmented parvoviral sequences have been detected in genome assemblies of *planarians* and *trematodes* (11, 12). Analyses of densovirus genome evolution and systematic characterization of integration patterns across Platyhelminthes lineages remain limited. This stands in contrast to the more extensively studied integration mechanisms of the vertebrate-infecting Parvovirinae, highlighting a significant gap in our understanding of invertebrate endogenous viral elements (EVEs).

The integration of non-retroviral viruses, including double-stranded DNA (dsDNA), single-stranded DNA (ssDNA), double-stranded RNA (dsRNA), and single-stranded RNA (ssRNA) viruses, as EVEs into host genomes is increasingly recognized as a widespread phenomenon (13–15). Non-retroviral viral genomes can integrate into the host’s chromosomal DNA through non-homologous recombination or by interacting with retroelements within the cell (16–18). Notably, densoviruses, as linear single-stranded DNA viruses, are no exception. Endogenization events have been documented not only for vertebrate-infecting Parvovirinae but also for Densovirinae in diverse invertebrate lineages (7, 19). The genomes of certain insects and other invertebrates contain sequences derived from densovirus, including genes that encode the NS1 protein. NS1 is a nonstructural protein essential for densovirus replication, and its gene sequence has been found in host genomes, indicating that these viruses once existed in an endogenous form and suggesting that the conservation of genes such as NS1 may facilitate their retention by the host. As such, these integrated viral sequences can be regarded as genomic “molecular fossils” of ancestral viral infections that became fixed in the host germline. Among these, the endogenous NS1 domain serves as a particularly valuable marker for tracing long-term virus-host coevolutionary dynamics. This approach is strongly motivated by the finding that the NS1 protein of the Densovirinae shares direct evolutionary ancestry with parvovirus-like NS1 domains identified in Platyhelminth genomes (20). This finding implies that densoviral genetic elements may be broadly retained in Platyhelminth genomes, potentially reflecting ancient host-virus interactions or ongoing cross-species transmission events. However, whether densovirus has endogenized in Platyhelminthes remains unresolved. Notably, sequences belonging to Platyhelminth parvovirus could constitute a common ancestor of parvoviruses, and phylogenetic evidence supports long-term codivergence between parvoviruses and their hosts (20, 21). If densoviruses also coevolved with their Platyhelminth hosts, host divergence times could serve as molecular clock calibration points to date ancient viral invasion events (22).

Approximately 80% of Platyhelminthes are obligate parasites, deriving nutrients from host organisms through ectoparasitism or endoparasitism. Medically significant taxa, such as *trematodes* and *cestodes*, exhibit complex multi-host life cycles, often involving aquatic intermediate hosts (such as snails) and vertebrate definitive hosts (including humans and livestock). Transmission routes primarily include ingesting contaminated water or undercooked meat containing the larval stage (23). The ecological complexity of these parasites is amplified by their high pathogenic potential. Densoviruses induce severe epizootics in arthropod hosts through lethal systemic infections (24). Separately, chronic inflammation, parasite-derived products, and physical damage are examples of interactions between parasites and hosts. These interactions, along with their extensive effects on chromosomes and cell fate, result in changes in cell growth, proliferation, and survival, which in turn cause and support malignant tumors (25). The convergence of high prevalence in human populations and livestock epidemics, with annual losses in agricultural productivity and healthcare costs, imposes substantial economic burdens (26, 27). However, the potential role of EVEs, such as those derived from densoviruses, in shaping their evolutionary history or biological traits remains entirely unexplored. Systematically investigating these integrated “molecular fossils” in Platyhelminthes not only illuminates ancient virus-host interactions but may also provide novel evolutionary contexts for understanding parasite biology.

The evolutionary interplay between densoviruses and their Platyhelminth hosts remains poorly characterized. While densovirus is known to infect arthropods, its potential integration into Platyhelminth genomes as EVEs has not been systematically investigated. This study aims to characterize the interaction between densoviruses and their Platyhelminth hosts through the following objectives: (i) to investigate whether the conserved NS1 helicase domain has been endogenized as viral fossils (EVEs) in Platyhelminth genomes, providing evidence of historical densovirus integration events; (ii) to reconstruct long-term evolutionary trajectories of densovirus and calibrate the timing of endogenization events based on host divergence times; and (iii) to explore the long-term evolutionary dynamics between densoviruses and Platyhelminth hosts.

## MATERIALS AND METHODS

### Genome screening and viral sequence identification

As of November 2024, a total of 126 Platyhelminth genome data sets were retrieved from NCBI (https://www.ncbi.nlm.nih.gov/datasets/genome) using the keyword “Platyhelminthes.” A local database containing all available Parvoviridae protein sequences downloaded from GenBank was constructed. The Platyhelminth genome data sets were then compared against the local database using the BLASTx program built into DIAMOND v2.0.1543 (28). Sequences with significant hits to NS1, as determined by the parameters “-f 6 -k 1 -e 0.00001,” were retained. To identify the NS1 open reading frame (ORF), the following steps were performed in Geneious Prime v2024 (https://www.geneious.com) (29): (i) sequences from the genomic data set were imported into Geneious Prime v2024. Based on the alignment results, nucleotide sequences containing NS1 were extracted. The "Find" function in Geneious Prime v2024 was then used to locate the insertion sites of these nucleotides within the sequence, with the search configured to interpret ambiguity codes in both the query and target sequences and to include the reverse-complement strand. (ii) Open reading frames (ORFs) were predicted with the ORF Finder tool (minimum length: 700 nucleotides; genetic code: Platyhelminthes mitochondrial; start codon: ATG) (30). Then, find the ORF containing this NS1 segment, and copy the ORF translation. (iii) The translated sequences were analyzed using BLASTP (31) against the NCBI non-redundant (NR) protein database (32). The top hit, identified as the cluster representative sequence, was examined for its percent identity and query coverage to confirm that the translated sequences encode the NS1 protein and its characteristic structural features.

### Phylogenetic analysis

To infer evolutionary relationships among densovirus NS1 proteins, NS1 protein sequences of Parvoviridae and densovirus were retrieved from NCBI GenBank as reference sequences. Sequences shorter than 400 amino acids (aa) were excluded to minimize alignment artifacts. Alignments were generated using Clustalw in MEGA-X v10.1.8 (33) with default parameters (gap opening penalty = 10, gap extension penalty = 0.2, BLOSUM matrix). The resulting alignments were then manually trimmed to remove poorly aligned regions, which were defined as those with a consensus sequence identity below 50%, to enhance the quality for phylogenetic analysis. A Markov chain Monte Carlo (MCMC) analysis was conducted using MrBayes v3.2.3 (34) with two independent runs of four chains each, each run sampling every 50 generations for 1 million generations. Convergence was assessed by ensuring that the average standard deviation of split frequencies fell below 0.01, the potential scale reduction factor (PSRF) approached 1.0 for all parameters, and the effective sample size (ESS) for each parameter exceeded 200. The first 25% of sampled generations were discarded as burn-in. The generated phylogenetic trees were annotated and visualized using ChiPlot (https://www.chiplot.online) (35). All viral NS1 protein sequences were also aligned using MAFFT v7.450 (36) with the auto-selected strategy (L-INS-I for < 200 sequences; FFT-NS-2 for larger data sets) and multi-threading enabled (--thread −1 to utilize all available physical cores). The Sequence Demarcation Tool (SDT) v1.3 (37) was employed to compute pairwise p-distances and generate a color-coded distance matrix between the identified densovirus sequences and the reference sequences.

### Timescale phylogenetic tree analysis

Divergence times for Platyhelminth hosts were retrieved from TimeTree (http://www.timetree.org) (38, 39), a curated database integrating molecular and fossil-based estimates. To estimate the divergence times of the viral NS1 sequences, a relative timescale (RelTime) phylogenetic tree was constructed in MEGA-X (40) using the maximum likelihood (ML) method under the JTT model (41, 42). The tree was calibrated using well-established divergence times of their Platyhelminth hosts obtained from the TimeTree database. Our search for calibration points among the 10 Platyhelminth species and reference sequences was limited. Due to the lack of reliable divergence time estimates in the TimeTree database for the other seven Platyhelminth species and the various reference sequences (such as barn owl, *Bactericera trigonica*, bat, rhesus monkey, and swine), only three calibration points were available and used in the analysis. These constraints included the divergence between *Echinococcus multilocularis* and *E. granulosus* (11 MYA), the stem age of the *Taeniidae* family (11 MYA, calibrated using *E. multilocularis* and *Taenia asiatica*), and the maximum age of the *Arthropoda* phylum (692 MYA, based on *Aedes aegypti*). Times were not estimated for outgroup nodes because the RelTime method uses evolutionary rates from the ingroup to calculate divergence times and does not assume that evolutionary rates in the ingroup clade apply to the outgroup. Other species were excluded from the final analysis as their divergence time data were not available in the TimeTree database (43–45).

### Flanking sequence analysis

To confirm the chromosomal integration of endogenous NS1 sequences in Platyhelminth genomes, we performed comprehensive flanking sequence analysis. We identified 88 sequences containing NS1 homologs from 13 Platyhelminth reference genome assemblies (e.g., GCF_000237925.2, GCF_000715545.1, GCF_000524195.1, and GCF_000469625.1). Putative NS1 coding regions were identified based on start codon (ATG) positions and sequence homology. For each potential NS1 integration site, we extracted 300 bp flanking sequences upstream and downstream of the predicted NS1 coding region. Sequence composition analysis included GC content calculation and characterization of the flanking region. Statistical comparisons were implemented in R v4.4.3 through the tidyr, dplyr, and ggplot2 packages (46).

### Prediction of potential genome recombination events

Recombination events were detected using the Recombination Detection Program v4.39 (RDP4) (47). Recombination analysis was performed on a data set comprising 88 sequence alignments, including alignments of reference strains and LWMJ02000172.1, all of which included the NS1 gene and its flanking sequences. Additionally, reference sequences from Platyhelminthes closely related to LWMJ02000172.1 were identified using BLASTP. All alignments were scrutinized with seven detection algorithms (RDP, GENECONV, BootScan, MaxChi, Chimaera, SiScan, and 3Seq) to identify recombination signals. A recombination event was considered statistically significant if it was detected by at least three different methods with a corrected *P*-value of less than 0.05.

### Prediction of spatial structure

The tertiary structure of densovirus NS1 proteins with different Platyhelminth hosts was predicted using AlphaFold3 (https://alphafoldserver.com) (48). High-quality models were selected based on pTM and ipTM scores, with thresholds set at pTM > 0.5 for the overall complex fold and ipTM > 0.8 for a high-confidence protein-protein interface (49). Predicted structures and aligned structures were visualized in PyMOL v4.6.0 (https://www.pymol.org). To accurately identify the corresponding ATPase and effector domains within the structures, a screening method based on a spatial distance threshold of 5 Å was employed, and structural similarity was quantified by calculating the root-mean-square deviation (RMSD) of Cα atoms. We defined an RMSD ≤2.0 Å as highly similar, 2.0–3.0 Å as moderately similar, and >3.0 Å as significantly divergent (50).

### Statistical analysis

Differences among the three clades were assessed by one-way ANOVA, with post-hoc Tukey HSD tests for pairwise comparisons. All analyses were conducted in R v4.4.3 at a significance level of α = 0.05.

## RESULTS

### Overview of the Platyhelminth genome sequence

**Fig 1:**
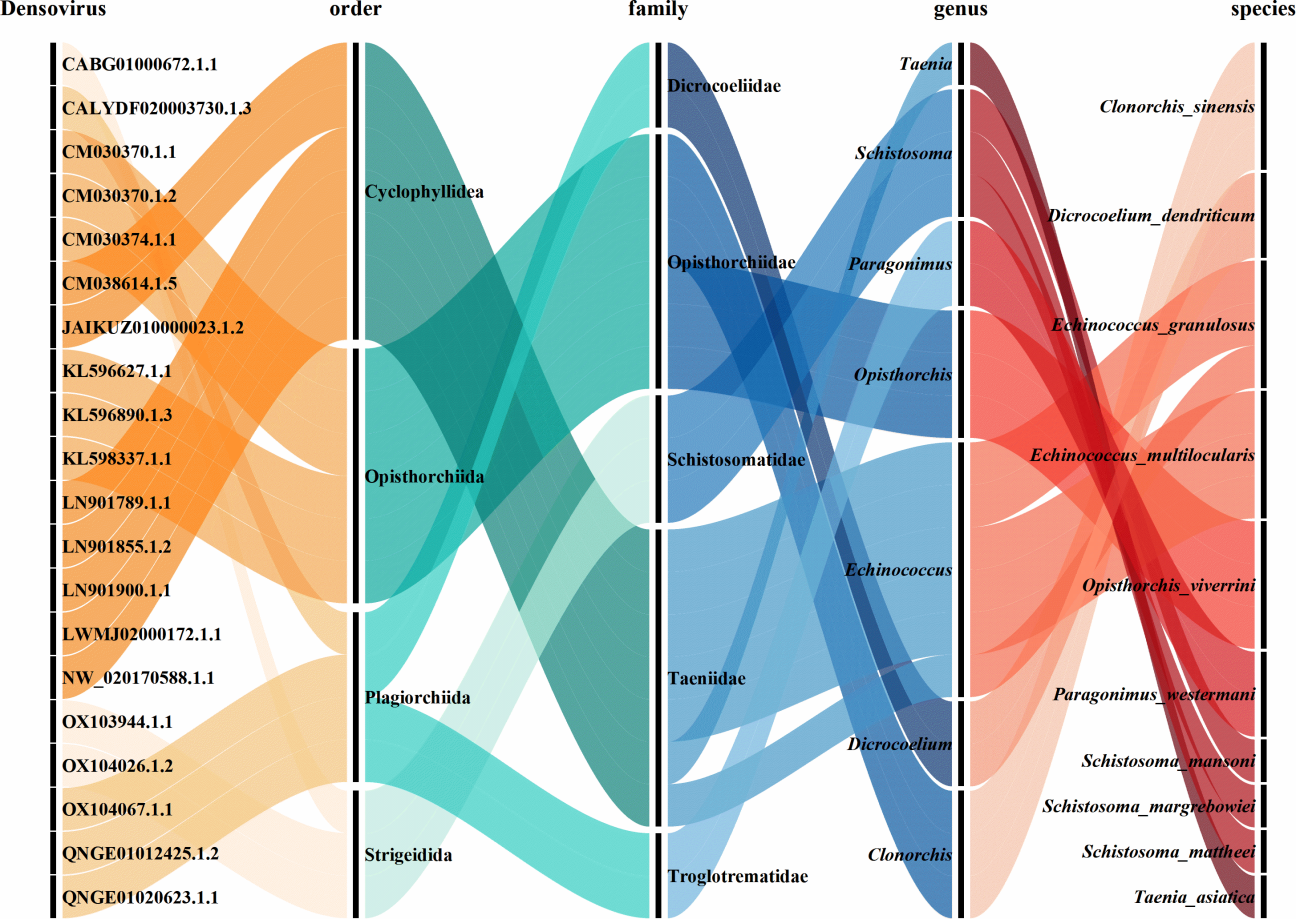
The orders, families, genera, and species of the Platyhelminth hosts of the representative densoviruses. An alluvial plot generated in Origin shows the flow across taxonomic hierarchies, including 20 sequences, four orders, five families, seven genera, and 10 species.

**Fig 2:**
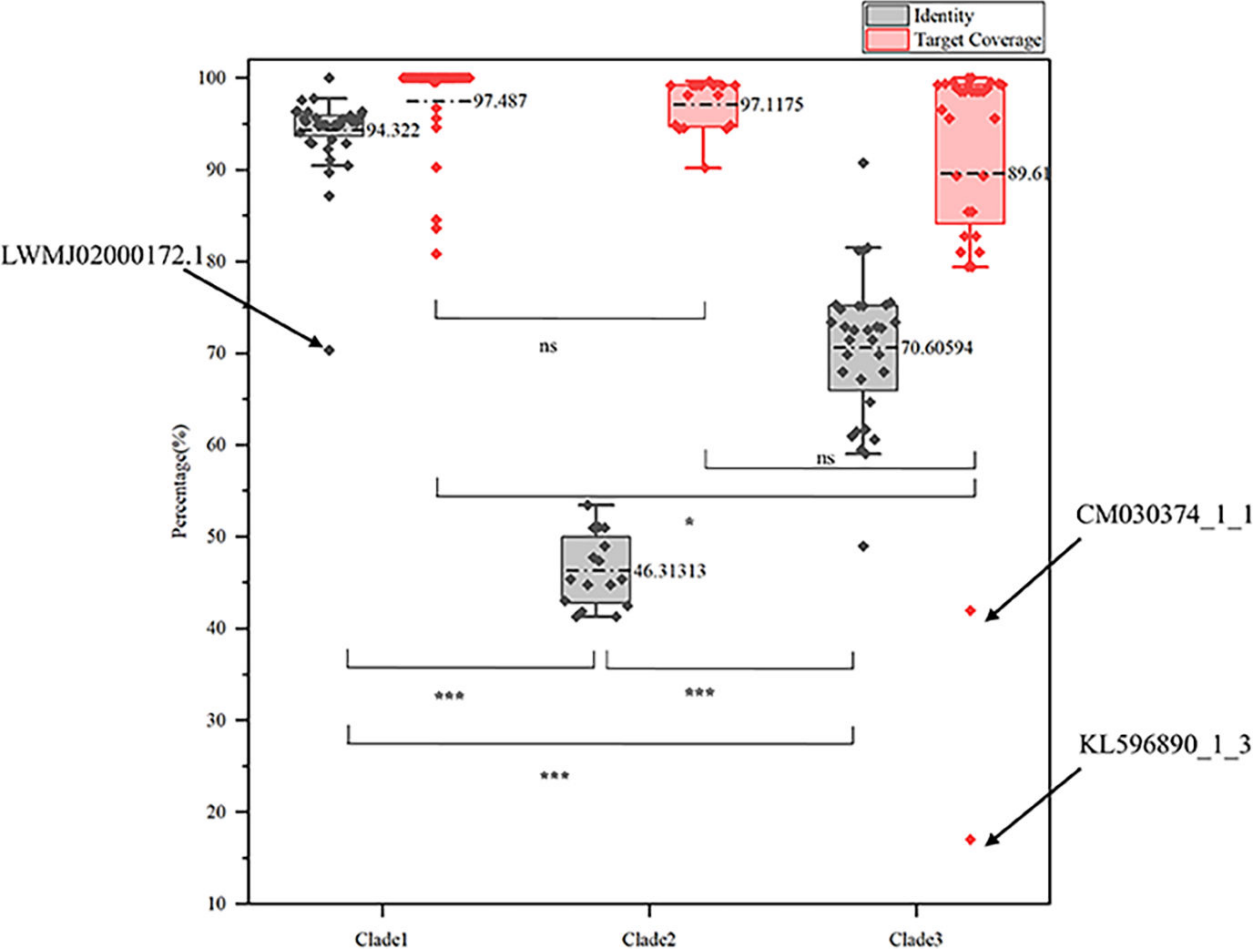
Amino acid identity and target coverage of different clades. Boxplot showing the identity and target coverage of the NS1 conserved domains relative to known viruses. Values represent percentage scores. The data represent mean values. Pairwise comparisons between the three groups: *P < 0.05, ***P < 0.001; ns, not significant.

**Fig 3:**
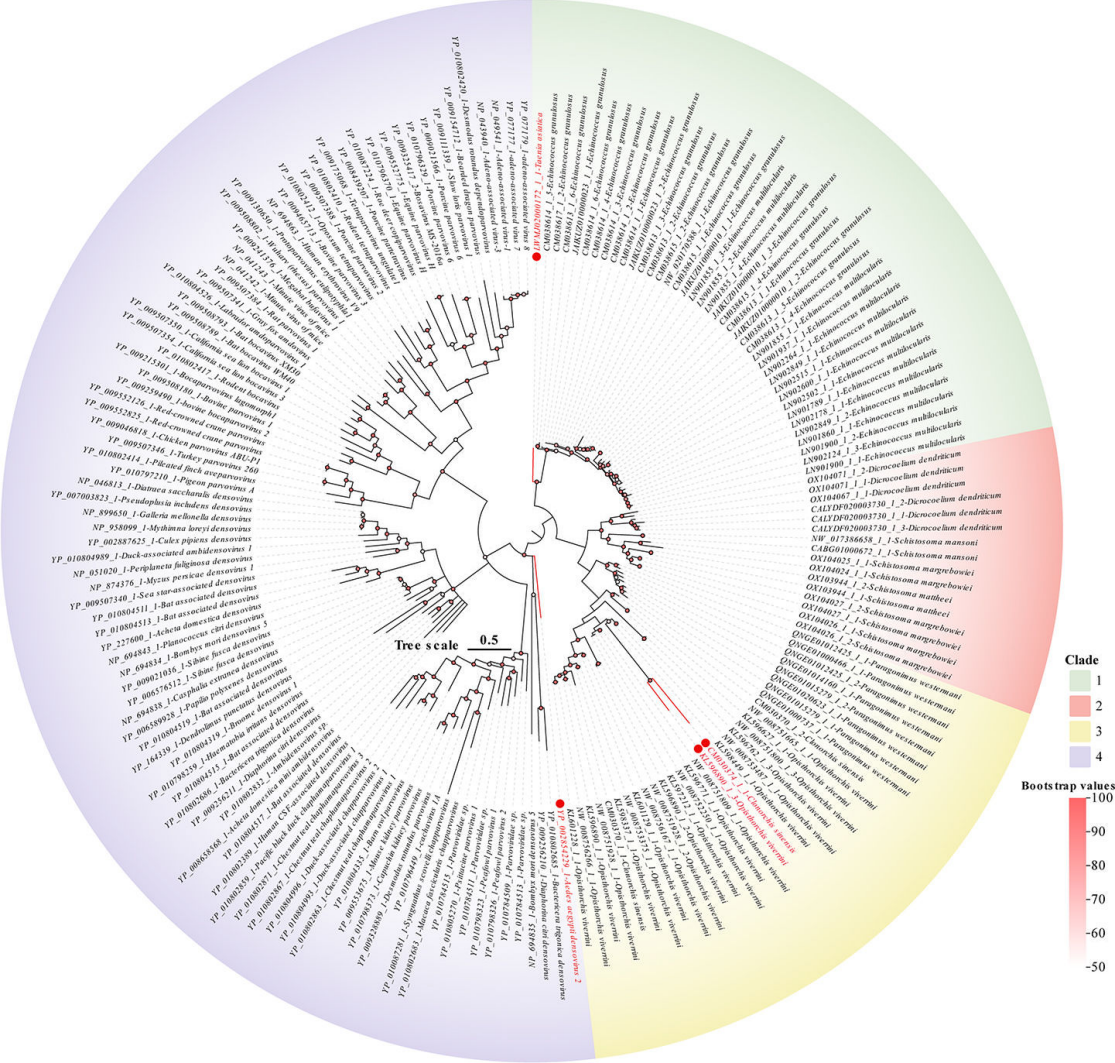
Phylogenetic relationship of Parvoviridae. A phylogenetic analysis tree was constructed based on the NS1 protein of Parvoviridae. The red dots represent *unclassified Parvoviridae*, and the black dots represent the *Penstyldensovirus genus. Taenia asiatica* (LWMJ02000172.1) forms a divergent clade. The scale bar indicates the number of amino acid substitutions per site.

We screened 126 Platyhelminth genome data sets retrieved from NCBI (Table S1). Following BLASTP screening against the NCBI NR database, we detected 88 NS1 protein sequences in 13 Platyhelminth genomes, including three pairs of genomes with the same species name. Sequence redundancy analysis revealed 15 duplicated combinations of entries, in which the NS1 ORF proteins were 100% identical (e.g., identical sequences in CM038614.1, JAIKUZ010000023.1, and CM038613.1; NW_017386658.1 and CABG01000672.1). After removing the duplicates, 67 NR NS1 sequences were retained. NS1 was identified in 10 species across seven genera (Fig. 1), with *Echinococcus granulosus* (25.0% of the total 88 NS1 protein sequences) and *Opisthorchis viverrini* (23.9%) as the predominant hosts. NS1 proteins exhibited a mean length of 648 amino acids (Table S2). The widespread presence of densoviruses across diverse Platyhelminthes lineages (e.g., *Schistosoma*, *Taenia*) suggests recurrent historical infections, potentially linked to host-parasite coevolution. Interestingly, BLASTP analysis revealed distinct levels of sequence identity and target coverage across the three clades (Fig. 3). Descriptive statistics revealed distinct levels of NS1 sequence identity across the three clades: Clade 1 (mean = 94%, SD = 5.4), Clade 2 (mean = 46%, SD = 4.2), and Clade 3 (mean = 70%, SD = 9.1). A one-way ANOVA demonstrated that these differences were highly statistically significant (*P* < 0.001). Subsequent post-hoc analysis with Tukey’s HSD test confirmed that all pairwise comparisons between clades were significant (all *P* < 0.001), solidifying that Clade 1, Clade 2, and Clade 3 represent three genetically distinct groups with significantly different levels of sequence identity. The one-way ANOVA revealed significant differences in target coverage among the three clades (*P* = 0.011). Descriptive statistics showed mean target coverage of 97% for Clade 1, 97% for Clade 2, and 89% for Clade 3. Tukey’s HSD post-hoc test identified a significant difference between Clade 3 and Clade 1 (*P* = 0.012), while the differences between Clade 2 and Clade 1 (*P* = 0.993) and between Clade 3 and Clade 2 (*P* = 0.084) were not statistically significant. Two outliers (KL596890_1_3, CM030374_1_1) exhibited low target coverage (<50%) (Fig. 2), potentially reflecting degraded or taxonomically divergent endogenous elements (14, 15).

### Phylogenetic analysis

To resolve the taxonomic affiliation of densoviral NS1 sequences identified in Platyhelminth genomes, we constructed a phylogenetic tree using 183 amino acid sequences of NS1, including 95 exogenous (7, 51) reference sequences from the Parvoviridae and densoviruses downloaded from RefSeq and 88 NS1 sequences from this study (Fig. 3). The phylogenetic analysis revealed that the endogenous NS1 sequences from Platyhelminthes, along with some exogenous references, formed several distinct clades. Specifically, the endogenous sequences clustered into two primary groups: one that includes viruses from the genus *Penstyldensovirus* (*n* = 30), and another that is currently not assigned to any established genus (*n* = 58) (Table S3). In the phylogenetic tree, endogenous NS1 sequences in Platyhelminthes formed a well-supported cluster, separate from exogenous densoviruses. This clear separation provides robust evidence for their ancient endogenization within the Platyhelminthes lineage, and their deep divergence suggests a prolonged history of host-virus coevolution, with the endogenous lineages evolving independently from their contemporary exogenous counterparts. Moreover, according to ICTV guidelines for Parvoviridae taxonomy, species demarcation requires >85% amino acid identity in the NS1 protein with >80% coverage, while genus-level classification necessitates ≥35–40% amino acid identity between any members (52). In Clade 1, *Taenia asiatica* (LWMJ02000172.1) forms a distinct branch and exhibits less than 75% sequence identity to all other members. This stands in clear contrast to the high identities (>75%) among all other sequences in Clade 1, suggesting that it likely represents a new species. We also observed that amino acid identity generally decreases with increasing evolutionary distance (1).

### Distance matrix analysis of densovirus NS1 sequences

**Fig 4:**
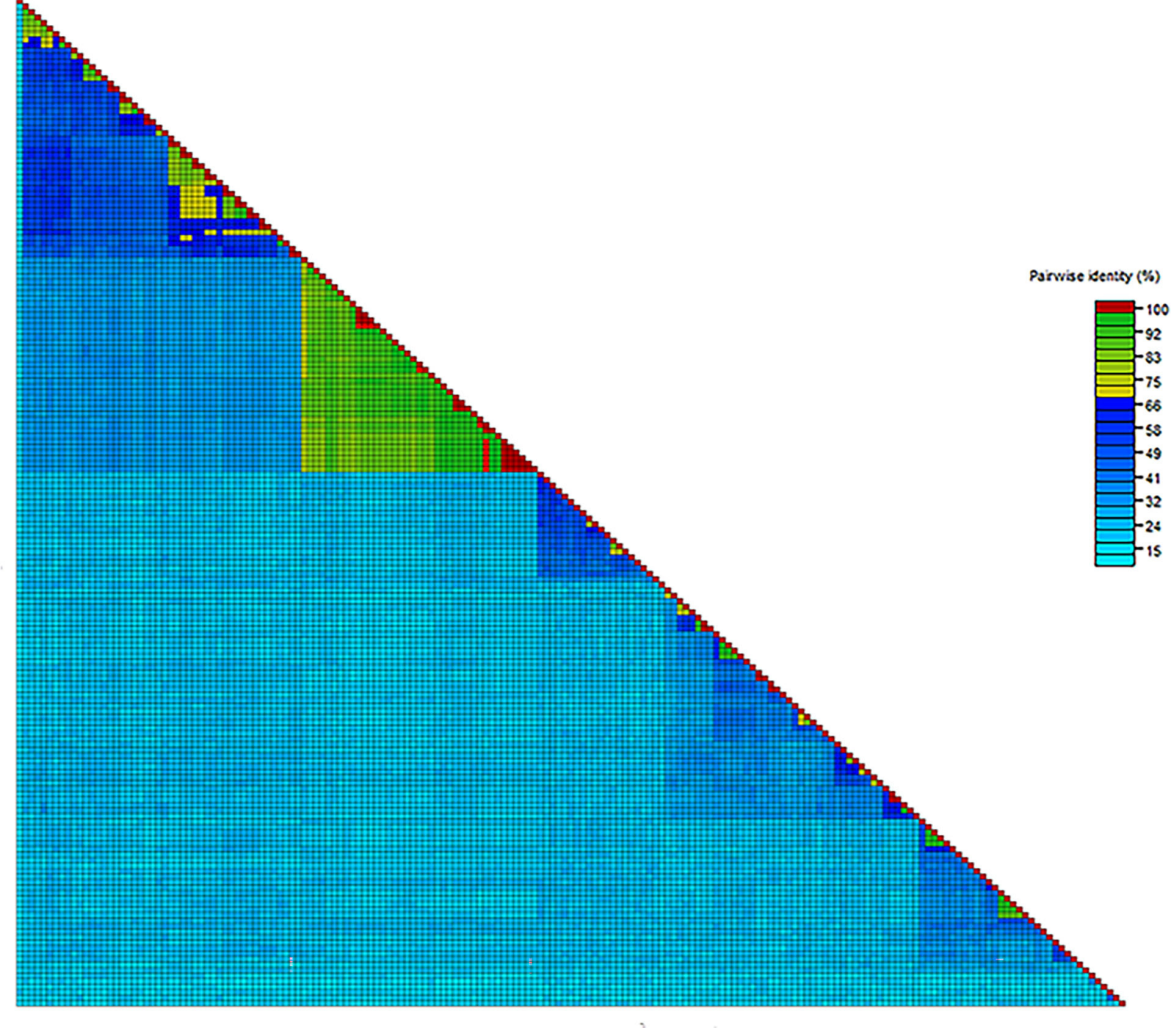
Distance matrix analysis of Parvoviridae. Pairwise sequence comparison was performed with NS1 protein amino acid sequences.

According to the distance matrix data, pairwise alignment of densovirus NS1 sequences revealed that similarity was notably high in *Echinococcus granulosus* and *Echinococcus multilocularis* (73.40–100.0%), with paralogous sequences (e.g., LN901900.1 vs LN902124.1) exhibiting 100% identity despite distinct amino acid compositions, suggesting selective retention of critical functional domains. Near-identical variants in *E. granulosus* (e.g., CM038613_1_2 vs JAIKUZ010000023_1_2: 99.60%) further underscored lineage-specific conservation. Similarly, *Paragonimus westermani* (mean similarity: 77.63%; range: 68.20–90.70%), *Dicrocoelium dendriticum* (mean similarity: 74.06%; range: 61.50–91.40%), and *Opisthorchis viverrini* demonstrated robust intraspecific sequence homogeneity. Pairwise alignment revealed a broad spectrum of sequence similarity among exogenous reference sequences, ranging from 13% to 100%. However, interspecific divergence was substantial, as exemplified by *Taenia asiatica* (LWMJ02000172.1), which showed limited similarity (≤25.30%) with other species. The distance matrix visualization further supported this interspecific divergence, with distinct clustering patterns indicating higher sequence conservation within species than between species (Fig. 4). These findings highlight intraspecific conservation versus interspecific divergence, which aligns with a host-virus codivergence model wherein densovirus lineages evolved independently within distinct host taxa.

### Evolutionary timeline of densovirus-Platyhelminthes codivergence

**Fig 5:**
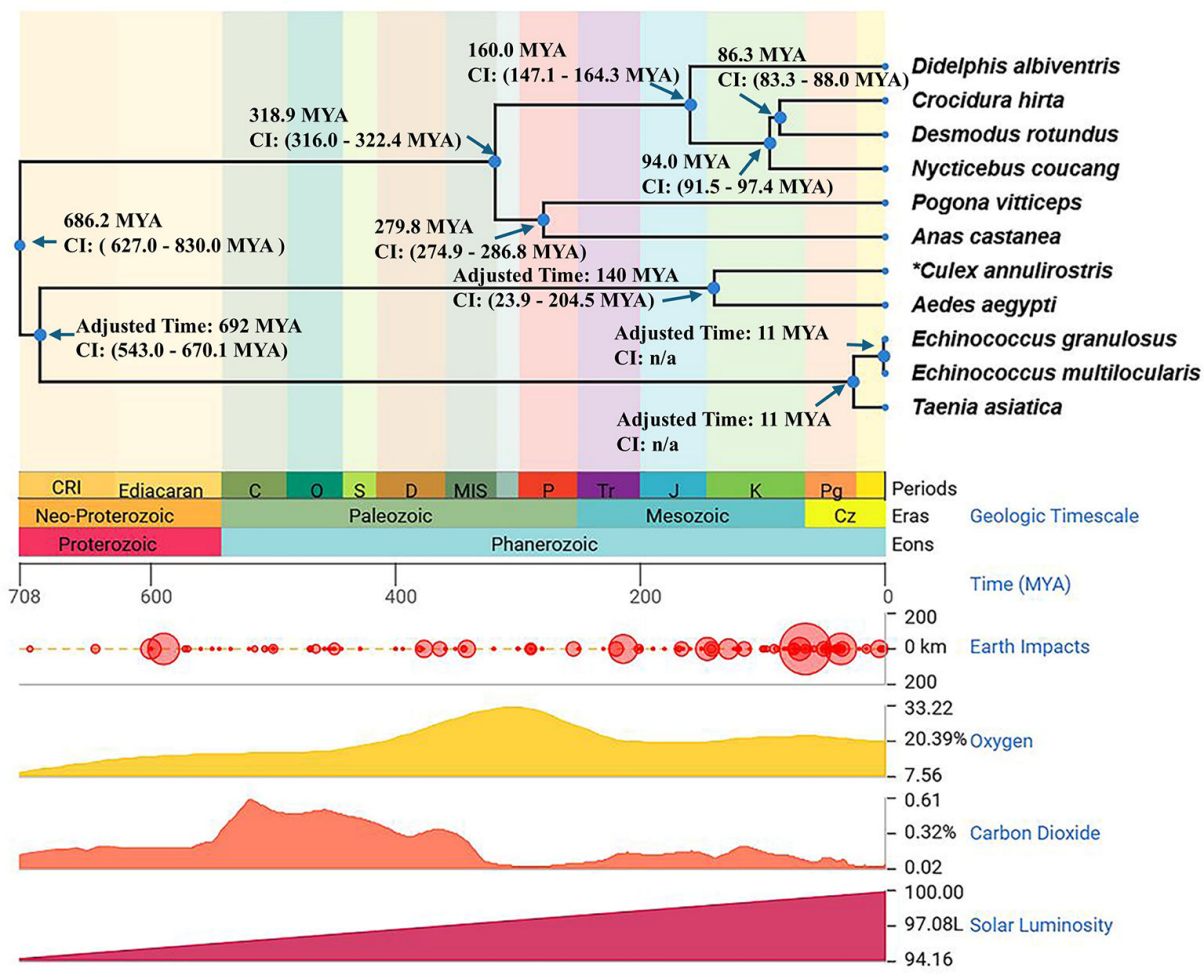
Timescale phylogenetic tree for hosts. The timescale phylogenetic tree depicts the time of divergence for the hosts. The time is displayed in geological timescale (periods, eras, and eons), millions of years ago (MYA), Earth impact, oxygen, carbon dioxide, and solar luminosity measurements. Adjusted times represent the accurate measurement. All other times are estimates.

**Fig 6:**
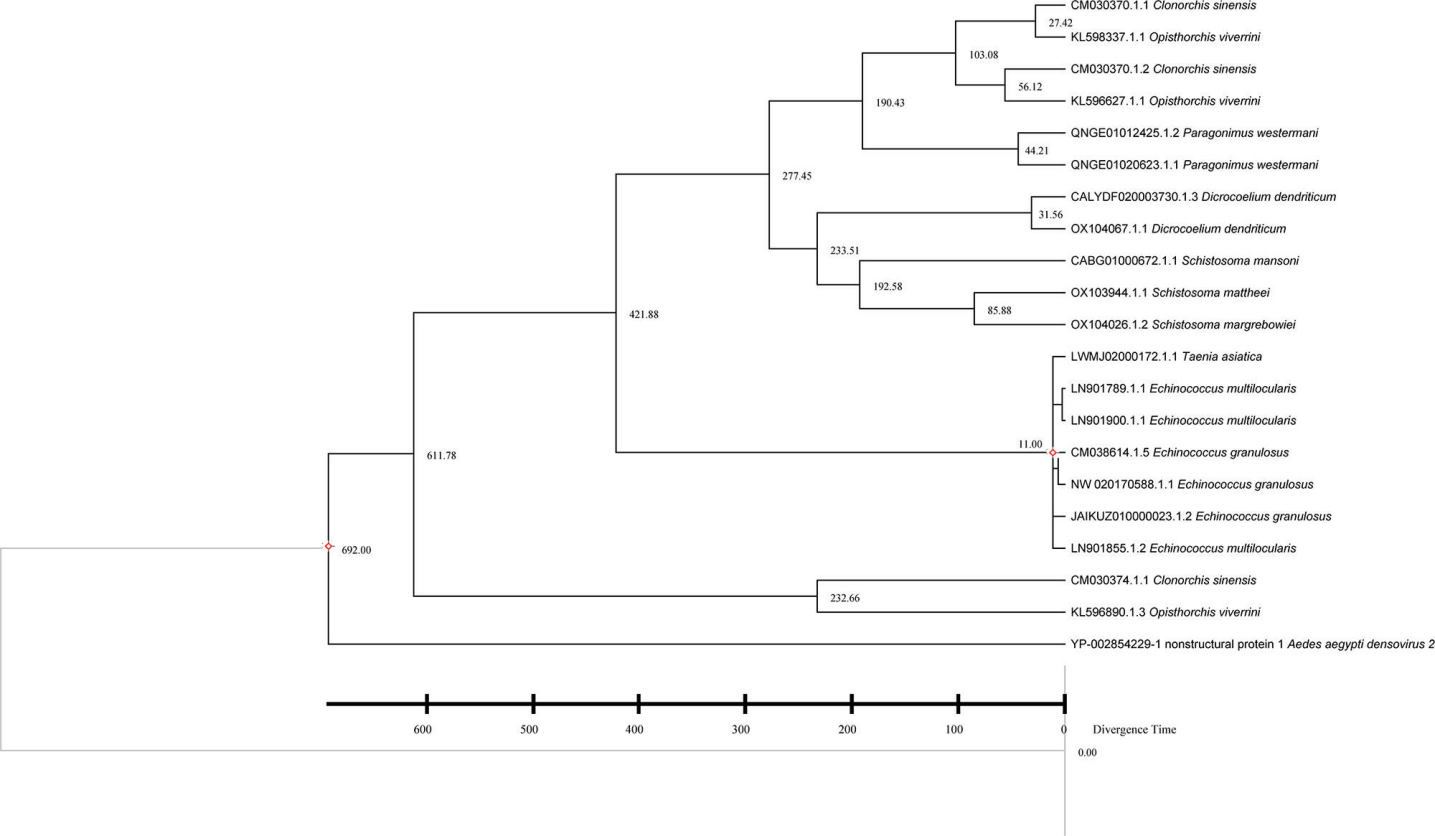
Relative timescale (RelTime) phylogenetic tree for parvo-NS1 domain. The relative timescale (RelTime) phylogenetic tree depicts the divergence time for the densovirus parvo-NS1 domains detected from Platyhelminths. The red diamonds represent the calibration of the phylogenetic tree using adjusted times obtained from TimeTree.

We have obtained evidence that hosts and viruses have coevolved and sought to further illustrate the interrelated evolutionary history of densoviruses and their respective hosts, tracing the timing of the independent evolution of Platyhelminthes and viruses. The integration age of densoviruses can be inferred from the divergence time of their Platyhelminth hosts. Timescale phylogenetic tree for hosts revealed that densovirus diverged from exogenous *Aedes aegypti densovirus* (YP_002854229.1) 610 MYA (adjusted to 692 million years) (22), and other exogenous *parvoviridae* reference sequences diverged from the endogenous densovirus lineages at 686.2 (CI: 627.0–830.0) MYA, indicating an ancient split between arthropod- and Platyhelminth-infecting densovirus, and also suggesting that the endogenization of densovirus in Platyhelminth hosts may have occurred approximately at 692 (CI: 543.0–670.1) MYA. Moreover, the two divergence events are the *Taenia-Echinococcus* split (25.6 MYA, adjusted to 11 MYA) and the *E. granulosus-E. multilocularis* split (1.65 MYA, adjusted to 11 MYA) (53) (Fig. 5). The high degree of NS1 sequence conservation between *E. granulosus* and *E. multilocularis* is consistent with their recent divergence from a common ancestor. This suggests long-term purifying selection on replication-critical domains despite host speciation events. For taxa lacking TimeTree data (e.g., *Paragonimus westermani*, *Dicrocoelium dendriticum*), we inferred a relative timescale phylogenetic tree using three adjusted time points. Extrapolated divergence times (e.g., *Schistosoma-Dicrocoelium*: 233.51 MYA; *Clonorchis sinensis*, *Opisthorchis viverrini*, and *Paragonimus westermani* lineage: 190.43 MYA) align with Mesozoic host radiations, suggesting viral codivergence during the adaptive radiation of Platyhelminthes. *Clonorchis sinensis* (CM030374.1.1) and *Opisthorchis viverrini* (KL596890.1.3) diverged around 232.66 MYA, likely reflecting niche-specific pressures driving localized adaptive mutations (54) (Fig. 6; Fig. S1).

### NS1 integration evidence through flanking sequence analysis

**Fig 7:**
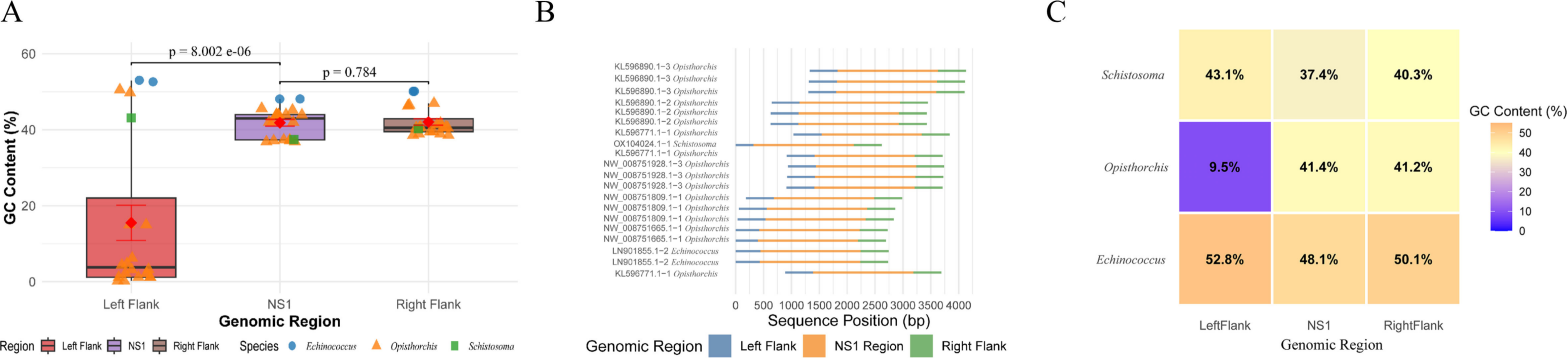
Flanking sequence analysis. (**A**) Bar plot shows the distribution of GC content in left flanking sequences, NS1 regions, and right flanking sequences. The *P*-value was calculated using a paired *t*-test. (**B**) Integration site analysis across 20 sequences. Colors indicate genomic regions (blue: left flank; orange: NS1; green: right flank). (**C**) The heatmap shows the average GC content in left flanking sequences, NS1 regions, and right flanking sequences across different species. Color intensity indicates GC content level.

Our flanking sequence analysis revealed compelling evidence for NS1 integration in platyhelminth chromosomes. We identified 20 distinct integration sites across multiple sequences by the conserved left flank-NS1-right flank structural organization (Fig. 7B), suggesting that NS1 sequences represent endogenous components integrated into platyhelminth chromosomes rather than transient contaminants.

Striking sequence composition differences were observed between NS1 regions and their flanking sequences. The NS1 regions maintained relatively stable GC content (mean = 41.84%, SD = 3.88%), while the left flanking sequences exhibited dramatically reduced and highly variable GC composition (mean = 15.52%, SD = 20.79%). This resulted in a significant 26.32% GC content (*P* < 0.001) disparity between left flanking sequences and NS1 regions (Fig. 7A). The pronounced compositional divergence indicates that NS1 elements have integrated into genomically diverse regions while maintaining their sequence signatures.

Species-specific integration patterns reveal both conserved and divergent evolutionary trajectories. The number of integration sites varied across species, ranging from 1 to 17 per species, with *Opisthorchis* genomes exhibiting the greatest diversity (17 distinct loci). These loci in *Opisthorchis* were characterized by exceptionally AT-rich left flanking sequences (mean GC content = 9.5%, range 0.2–50.47%) (Fig. 7C), demonstrating a tendency for integration into genomically distinct regions. The widespread distribution of these integrations across evolutionarily divergent taxa suggests that NS1 integration is an evolutionarily conserved genomic feature, rather than a result of species-specific accidental events.

The flanking sequences exhibited typical host genomic characteristics, including moderate GC content in the right flanking regions (38.52–50.1%), consistent with platyhelminth genomic composition. The structural integrity of the flank-NS1-flank organization across multiple independent genomic contexts provides compelling evidence that these elements represent bona fide EVEs that have become stable components of platyhelminth genomes.

### Recombination analysis

Reference sequences exhibiting identity (44.92%–70.34%) to LWMJ02000172.1 were screened using the BLASTP program. Recombination events were identified in a total of 76 sequences from nine species. The only exception was *Taenia asiatica*, where no recombination was found. To further validate this finding, LWMJ02000172.1 was tested against six additional homologous sequences, and the results showed no recombination in LWMJ02000172.1 (Table S4).

### Structural divergence of NS1 proteins across Platyhelminth hosts

**Fig 8:**
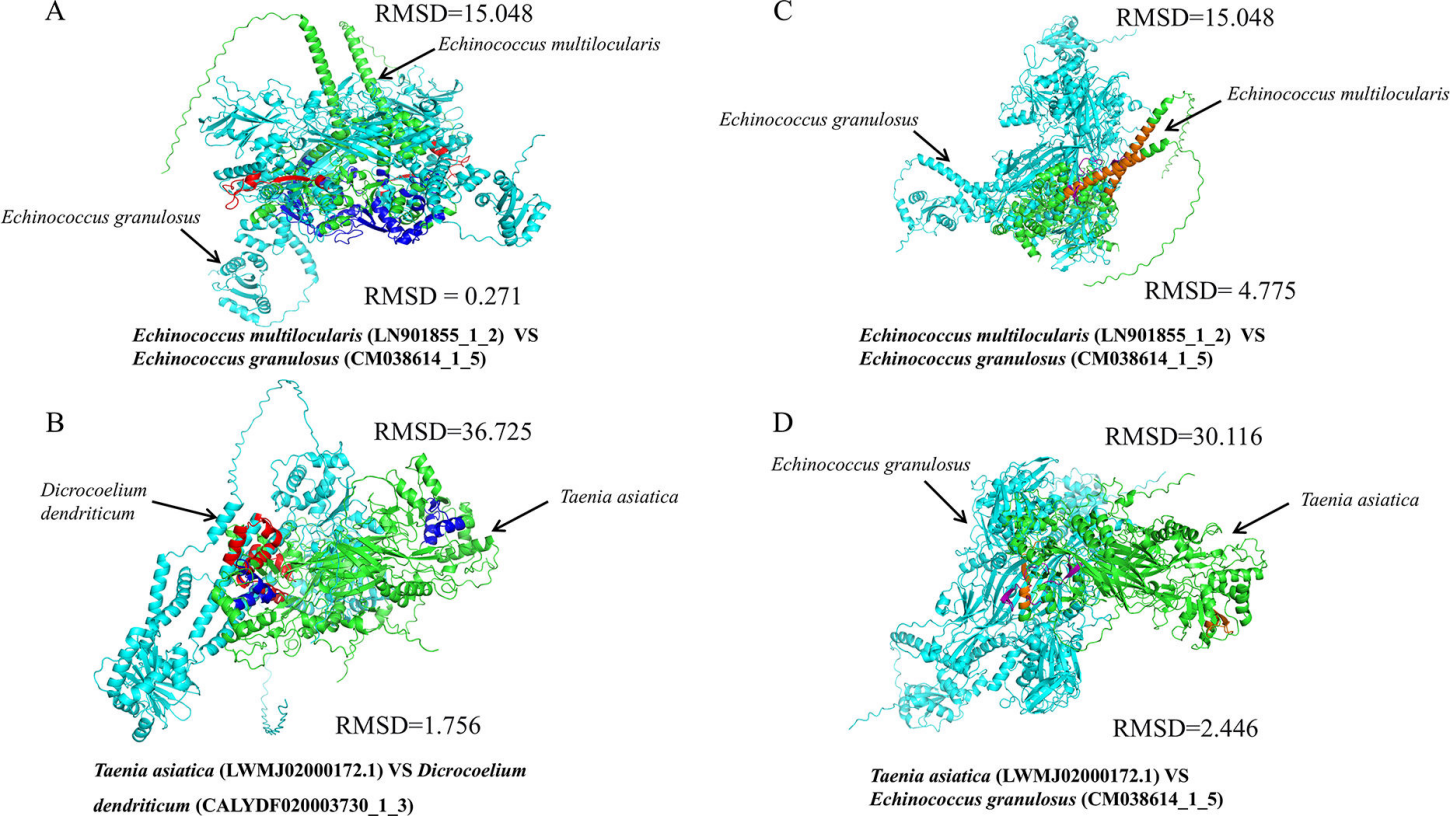
Viral NS1 protein structure alignment. Sequences were visualized and pairwise aligned using PyMOL software. RMSD indicates structural differences. (**A and B**) Structural alignment of the conserved ATPase domain. (**C and D**) Structural alignment of the effector domain. Top-right values in each panel indicate the global RMSD (Å) for NS1 protein alignment. Bottom-right values in each panel indicate the local RMSD (Å) for the corresponding functional domain alignment. Different structural regions are highlighted in distinct colors for clarity.

To assess functional divergence during Densovirinae evolution, we compared the tertiary structures of NS1 proteins from Platyhelminth lineages. A systematic structural comparison was performed on the functional proteins from four parasitic species: *Echinococcus multilocularis*, *Echinococcus granulosus*, *Taenia asiatica*, and *Dicrocoelium dendriticum*. Global structural alignment revealed substantial divergence in overall protein architecture among the different parasite species. The global RMSD between *E. multilocularis* and *E. granulosus* (closely related species) was 15.048 Å. This value increased to 30.116 Å between *T. asiatica* and *E. granulosus* and reached 36.725 Å between *T. asiatica* and *D. dendriticum* (distantly related species) (Fig. 8). These results indicate a clear positive correlation between evolutionary divergence and structural variation, with this variation being well beyond the 10 Å threshold commonly associated with functional conservation within protein superfamilies (55). In stark contrast, the local functional domains exhibited a high degree of structural conservation. Within the core functional region of the ATPase domain, the RMSD was 0.271 Å between *E. multilocularis* and *E. granulosus*, and 1.756 Å between *T. asiatica* and *D. dendriticum*, both values signifying a highly conserved structural architecture. For the effector domain, the RMSD was 4.775 Å between *E. multilocularis* and *E. granulosus*, and 2.446 Å between *T. asiatica* and *E. granulosus*, demonstrating a moderate to good level of conservation, albeit lower than that observed for the ATPase domain.

## DISCUSSION

The evolutionary history of endogenous densovirus NS1 proteins in Platyhelminthes is characterized by a dual signature: the long-term preservation of essential functional domains alongside widespread structural and sequence diversification in non-conserved regions. Statistical analysis of the three endogenous viral clades reveals diverse evolutionary patterns. In terms of sequence identity, all three clades show highly significant genetic divergence from each other (all *P* < 0.001), indicating they have evolved into three distinct genetic lineages. Regarding target coverage, both Clade 1 and Clade 2 maintain highly complete sequence structures (mean >95%) with no difference between them, whereas Clade 3 shows significantly lower coverage (88.4%, *P* = 0.012). These findings collectively support a compelling inference: these endogenous viral sequences have undergone significant adaptive radiation following prolonged co-evolution within their Platyhelminth hosts (51, 56). This work expands our understanding of densoviral diversity in invertebrate hosts. It reveals the extensive retention of densovirus NS1 in the genome of Platyhelminthes and the structural conservation of its core functional domains, providing molecular evidence for understanding virus-parasite coevolution.

Our phylogenomic analysis of densovirus NS1 sequences reveals profound evolutionary divergence between endogenous Platyhelminthes-integrated elements and exogenous parvoviruses. The clustering of Platyhelminthes-derived NS1 sequences on distinct phylogenetic branches provides strong support for their endogenization. This deep divergence aligns with patterns of host-virus coevolution. Moreover, based on the classification rules for Parvoviridae and the results of recombination analysis, we propose that LWMJ02000172.1 (from *Taenia asiatica*) represents a new species.

Distance matrix analysis showed that intraspecific conservation and pronounced interspecific divergence in densovirus NS1 sequences support a model of host-virus codivergence. The retention of near-identical paralogs likely reflects purifying selection on replication-critical domains (e.g., helicase, endonuclease), while lineage-specific divergence in variable regions suggests adaptive evolution under host-driven pressures.

Our flanking sequence analysis provides definitive molecular evidence for the chromosomal integration of NS1 sequences in platyhelminth genomes. The significant GC content disparity between NS1 coding regions and their flanking sequences (15.52% vs 41.84%) strongly suggests that these NS1 elements represent integrated viral sequences within host chromosomal contexts, rather than assembly artifacts or contaminating sequences. The identification of multiple integration sites within a single sequence indicates that NS1 elements have integrated at various genomic locations, potentially through distinct mechanisms or evolutionary events. The conservation of these integration patterns across multiple Platyhelminth species suggests that these integration events occurred early in evolution and have been maintained through vertical inheritance.

Our time-calibrated phylogenetic tree reveals an ancient split between arthropod- and Platyhelminth-infecting densovirus and also suggests that the endogenization of densovirus in platyhelminth hosts may have occurred approximately at 692 MYA, coinciding with early metazoan diversification (Ediacaran-Cambrian transition). The time of differentiation of other flatworms has also been inferred (e.g., *Schistosoma-Dicrocoelium*: 233.51 MYA; *Clonorchis sinensis*, *Opisthorchis viverrini*, and *Paragonimus westermani* lineage: 190.43 MYA). These time-calibrated data empirically validate virus-host codivergence over geological timescales.

Structural alignments reveal that the three-dimensional architectures of NS1 proteins from densoviruses infecting different Platyhelminth hosts exhibit significant divergence. During the evolution of parasitic functional proteins, core catalytic domains, such as the ATPase domain, are subject to intense selective pressure due to their crucial biological functions, resulting in extreme structural conservation. In contrast, non-core regions or functionally adaptable effector domains tolerate greater structural variation, allowing for adaptation to species-specific ecological niches. These patterns mirror EVE dynamics, where long-term host association drives functional optimization and genomic domestication.

### Conclusion

In conclusion, our findings clearly reveal a significant evolutionary pattern: “global divergence, local conservation.” This study provides evidence of densoviral endogenization in Platyhelminth hosts, advances our understanding of virus-host codivergence, and offers a framework for reconstructing paleoviral dynamics using endogenous “molecular fossils.

### Limitations

Based on our limited inferences from partial sequences and the sparse fossil record for Platyhelminthes in the TimeTree database, we cannot directly determine divergence times for flatworms. Therefore, the proposed evolutionary timeline should be viewed as a preliminary framework rather than a definitive chronology. Future expansions of flatworm genomic diversity and improved fossil calibrations will be essential to refine these estimates.

## Data Availability

The genomic data were downloaded from https://www.ncbi.nlm.nih.gov/datasets/genome/?taxon=6157. Further details are provided in Table S1.

## SUPPLEMENTAL MATERIAL

Figure S1 spectrum.03441-25-s0001.tif The original phylogenetic tree with bootstrap support values.

Tables S1 spectrum.03441-25-s0002.xlsx A data set of 126 platyhelminth genomes from NCBI, highlighting a subset of 13 genomes containing NS1 sequences, which includes 15 duplicate entries defined by identical NS1 ORF sequences.

Tables S2 spectrum.03441-25-s0003.xlsx The taxonomic classification (order, family, genus, and species) of the Platyhelminthes hosts harboring the Densovirus NS1 proteins.

Tables S3 spectrum.03441-25-s0004.xlsx The orders, families, genera, and species corresponding to NS1 sequences.

Tables S4 spectrum.03441-25-s0005.xlsx Recombination analysis of the 88-sequence data set and of LWMJ02000172.1 with its six homologous sequences.

ASM does not own the copyrights to Supplemental Material that may be linked to, or accessed through, an article. The authors have granted ASM a non-exclusive, world-wide license to publish the Supplemental Material files. Please contact the corresponding author directly for reuse.
