# Supplementary figures and images for: Evolutionary dynamics of endogenous densoviruses NS1 proteins reveal ancient codivergence with their Platyhelminth hosts
Source: Microbiol Spectr. 2026 Feb 27;14(4):e03441-25. doi: 10.1128/spectrum.03441-25 (PMC13055364; doi:10.1128/spectrum.03441-25)

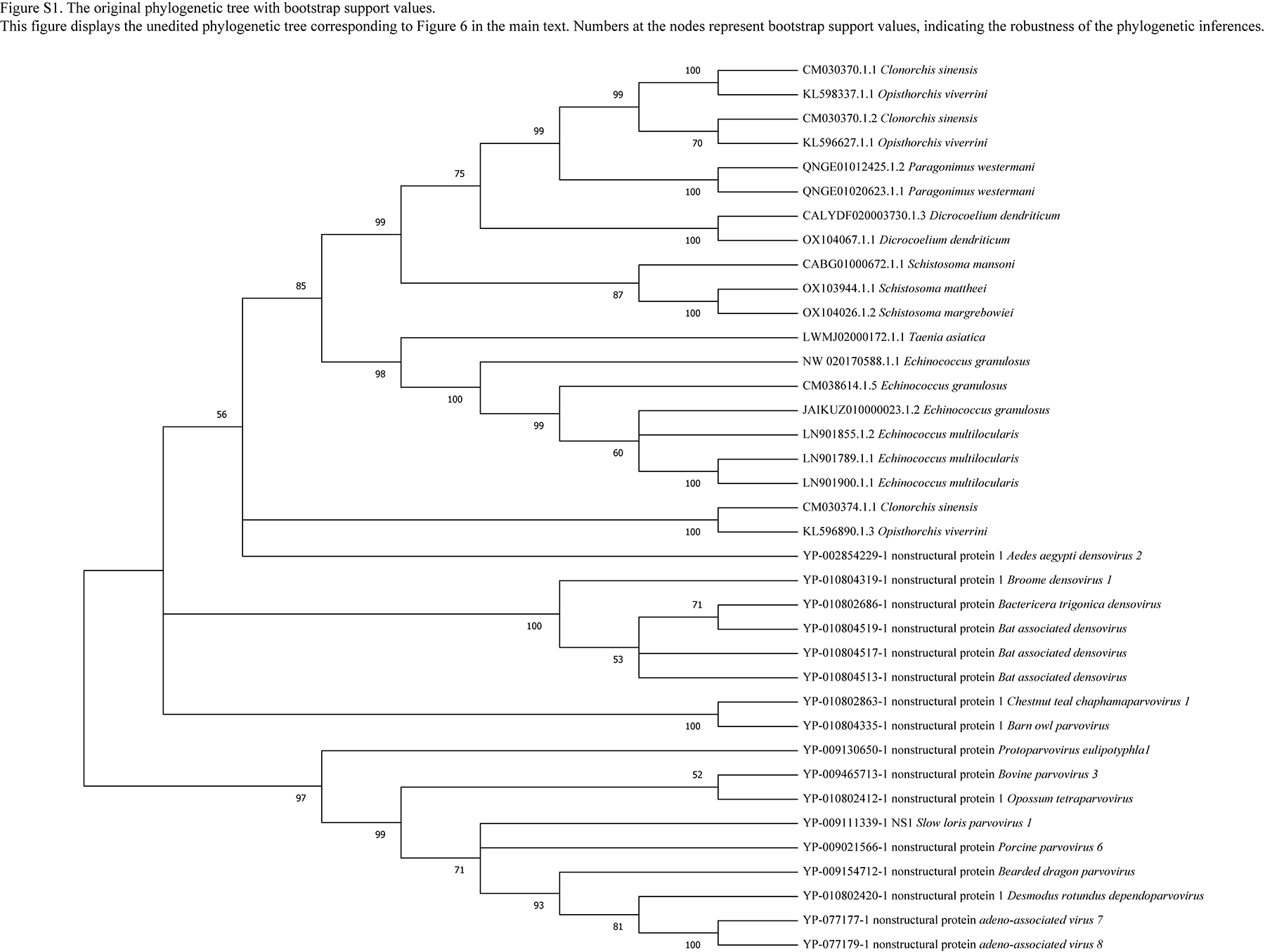

Supplement: Figure S1 — The original phylogenetic tree with bootstrap support values. [file spectrum.03441-25-s0001.tif]
